# Darier-White disease and Psychiatric disorders: A Case Report

**DOI:** 10.1192/j.eurpsy.2022.474

**Published:** 2022-09-01

**Authors:** R.L. Esteve, R. Sánchez-González, M. Campillo, E. Carrió

**Affiliations:** Institut de Neuropsiquiatria i Addiccions, Centre Emili Mira, Parc de Salut Mar, Department Of Psychiatry, Barcelona, Spain

**Keywords:** Darier Disease, schizophrénia, Neuropsychiatry

## Abstract

**Introduction:**

Darier-White disease (DD) is a rare genodermatosis of dominant autosomic inheritance characterized by the keratinization of epidermis, nails and mucous membrane. It leads to the formation of papules and brown hyperkeratotic plaques, mainly in seborrheic areas. The disease is associated with a mutation on the ATP2A2 gene, mapped in the 12q23-24 chromosome. There is known a relationship between DD and neuropsychiatric diseases, such as bipolar disorder, depression and schizophrenia.

**Objectives:**

To discuss the relationship between DD and neuropshychiatric disorders.

**Methods:**

We report the case of a patient with diagnosed schizophrenia, alcohol and cannabis dependence who presented skin lesions.

**Results:**

The physical exploration of our patient revealed cutaneous lesions and we pointed the diagnostic towards DD. Afterwards, a cross-consultation was done with the dermatology experts. During the physical exploration, the patient shows confluent hyperkeratotic papules, dominant on the sides and center of back and hands, together with nail injuries (see images). The diagnostic was confirmed through anatomic pathology. The patient was treated with 10 mg/day of Acitretin together with emollients twice a day, which improved the patient clinical status and signs. The patient remained stable at a psychiatric standpoint. After 3 years of treatment, the patient keeps the same medication but with a reduced dose of 5 mg/day, with a 70% decrease of the initial hyperkeratotic lesions.

**Figure:**
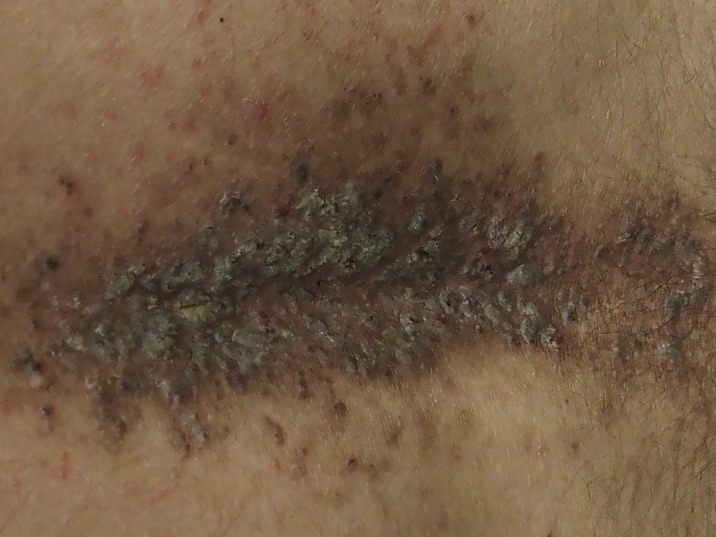

**Figure:**
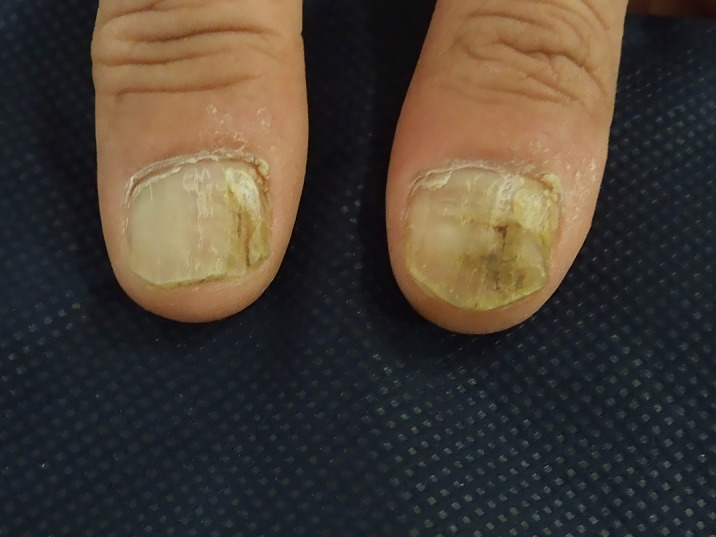

**Conclusions:**

Previous studies concludes that mutations in the ATP2A2 gene, in addition to causing DD, confer susceptibility to neuropsychiatric features.These case report highlight the need for clinicians to asses and recognize neuropsychiatric symptoms in DD.

**Disclosure:**

No significant relationships.

